# High frequency of exon 15 deletion in the *FANCA* gene in Tunisian patients affected with Fanconi anemia disease: implication for diagnosis

**DOI:** 10.1002/mgg3.55

**Published:** 2014-02-05

**Authors:** Ahlem Amouri, Faten Talmoudi, Olfa Messaoud, Catherine D d'Enghien, Mariem B Rekaya, Ines Allegui, Héla Azaiez, Rym Kefi, Ahlem Abdelhak, Sondes H Meseddi, Lamia Torjemane, Monia Ouederni, Fethi Mellouli, Héla B Abid, Lamia Aissaoui, Mohamed Bejaoui, Tarek B Othmen, Dominique S Lyonnet, Jean Soulier, Mongia Hachicha, Koussay Dellagi, Sonia Abdelhak, Tunisian Fanconi

**Affiliations:** 1 Department of Histology and Cytogenetics, Institut Pasteur de TunisTunis, Tunisia; 2 Laboratory of Biomedical Genomics and Oncogenetics, Institut Pasteur de Tunis, Tunis El Manar UniversityTunis, Tunisia; 3 Department of Tumour Biology, Institut CurieParis, France; 4 Haematology Department, Hedi Chaker Hospital, University of SfaxSfax, Tunisia; 5 Department of Haematology and Transplantation, National Bone Marrow Transplantation CentreTunis, Tunisia; 6 Department of Peadiatric Immuno-Haematology, National Bone Marrow TransplantationTunis, Tunisia; 7 Haematology Department, Aziza Othmana HospitalTunis, Tunisia; 8 Institut Curie, INSERM U830Paris, France; 9 Sorbonne Paris Cité, Université Paris DescartesParis, France; 10 Hôpital Saint-LouisParis, France; 11 Department of Pediatrics, CHU Hedi ChakerSfax, Tunisia; 12 Laboratory of Transmission, Immunology and Infection Control, Institut Pasteur de TunisTunis, Tunisia

**Keywords:** Exon 15 deletion, Fanconi anemia, founder haplotype, founder mutation, MLPA

## Abstract

Tunisian population is characterized by its heterogeneous ethnic background and high rate of consanguinity. In consequence, there is an increase in the frequency of recessive genetic disorders including Fanconi anemia (FA). The aim of this study was to confirm the existence of a founder haplotype among FA Tunisian patients and to identify the associated mutation in order to develop a simple tool for FA diagnosis. Seventy-four unrelated families with a total of 95 FA patients were investigated. All available family members were genotyped with four microsatellite markers flanking *FANCA* gene. Haplotype analysis and homozygosity mapping assigned 83 patients belonging to 62 families to the FA-A group. A common haplotype was shared by 42 patients from 26 families at a homozygous state while five patients from five families were heterozygous. Among them, 85% were from southern Tunisia suggesting a founder effect. Using multiplex ligation-dependent probe amplification (MLPA) technique, we have also demonstrated that this haplotype is associated with a total deletion of exon 15 in *FANCA* gene. Identification of a founder mutation allowed genetic counseling in relatives of these families, better bone marrow graft donor selection and prenatal diagnosis. This mutation should be investigated in priority for patients originating from North Africa and Middle East.

## Introduction

Fanconi anemia (FA) is one of the best defined inherited bone marrow failure (BMF) syndromes. It is usually inherited as an autosomal recessive trait, but in a small subset of FA cases it can be X-linked (Meetei et al. 2004). FA patients show marked clinical heterogeneity. Characteristic features include a progressive BMF and an increased predisposition to malignancy. Affected individuals may also exhibit one or more congenital/developmental abnormalities including abnormal skin pigmentation (e.g., café au lait spots), skeletal (e.g., radial ray anomalies), genitourinary (e.g., horseshoe kidney), and gastrointestinal (e.g., duodenal atresia) abnormalities (Soulier 2011).

A characteristic feature of FA cells is their hypersensitivity to DNA crosslinking agents such as Diepoxybutane (DEB) (Auerbach 2003) and Mitomycin C (MMC) (Cervenka et al. 1981). This feature is regularly used to distinguish FA patients from other aplastic anemia (AA).

FA is also a genetically heterogeneous disorder with at least 16 genes responsible of the known FA complementation groups (A, B, C, D1 [BRCA2], D2, E, F, G, I, J [BRIP1], L, M, N [PALB2], O [RAD51C], P [SLX4] and XP-F) (Soulier 2011; Alter and Kupfer 2002; Kashiyama et al. 2013).

The frequency of each complementation group has been shown to vary considerably according to the ethnic origin. FA-A is the most frequent group worldwide, accounting for 65% of all cases (Soulier 2011). In Tunisia, our previous studies on FA patients have shown that FA-A is the most encountered group with a frequency of 94%. It has been also reported that the majority of patients originated from southern Tunisia (Bouchlaka et al. 2003).

Furthermore, it was reported that almost 15–20% of mutations in *FANCA* gene are large deletions (Morgan et al. 1999; Castella et al. 2011). It has been frequently demonstrated that the majority of these deletions are due to a recombination between in cis-Alu-repeats (Castella et al. 2011). Screening for large deletions is usually performed by multiplex ligation-dependent probe amplification (MLPA) as an initial step in the mutational screening approach (Morgan et al. 1999).

The aim of this study was to extend the molecular study to a larger cohort for haplotype classification and to identify the mutation associated to this founder haplotype.

## Patients and Methods

### Patients

After written informed consent, 83 families including 95 FA patients were investigated. Among these families, 74 were unrelated.

Familial history and genealogical data were collected from patients and their families at the time of genetic questionnaire. When blood samples were sent from collaborating clinical departments, genealogical data were collected from clinical records or from the Tunisian Fanconi Anemia Registry (TFAR) (Hadiji Mseddi et al. 2012).

All patients were diagnosed as FA on the basis of clinical symptoms and/or family history and/or hypersensitivity to Mitomicyn C at a final concentration of 50 ng/mL in lymphocyte culture (Talmoudi et al. 2013).

### Genotyping test

All patients and their family members whenever available were genotyped with three or four markers flanking the *FANCA* gene (D16S3026, D16S3121, D16S3407, D16S303) as reported by Bouchlaka et al. (2003). The markers span an interval of about 142.88 kb and have the following order from centromere to telomere: D16S3026-D16S3121-D16S3407-D16S303. The D16S3407 is an intragenic marker located 16 kb upstream of the 5′ untranslated region of the gene.

Genotyping technique is based on a fluorescent-labeled universal M13 primer, which allowed analysis and subsequent genotyping with a high precision of the allele size using and automated DNA sequencer. Genotyping technique was based on the addition of a sequence to the 5′ end of the reverse primer of the microsatellite marker with no homology to the target genome.

Polymerase chain reaction (PCR) reactions were performed using the reverse primer, the forward primer and the M13 sequence: FAM-TGT AAA ACG ACG GCC AGT-3′. The PCR mix contained 0.8 *μ*mol/L of each forward and FAM-M13 primer and 0.08 *μ*mol/L of the reverse primer in a final 25 *μ*L reaction. Conditions of the PCR amplification are as follows: 94°C (5 min), then 35 cycles at 94°C (30 sec)/55°C (30 sec)/72°C (30 sec), and a final extension at 72°C for 30 min.

Subsequently, 1 *μ*L of the PCR product is added to 12 *μ*L of formamide and 0.5 *μ*L of ROX standard and run on an ABI prism 3130 DNA Genetic Analyzer (Applied Biosystems, Foster City, CA).

### Mutation screening by MLPA

MLPA analysis was used to detect large deletions or insertions in the *FANCA* gene. An MLPA kit for the *FANCA* gene, SALSA P031/P032, was used in this study (MRC-Holland, Amesterdam, Netherlands). Briefly, DNA was denatured for 5 min at 98°C, 3 *μ*L of the probe mix was added, after which the mixture was heated for 1 min at 98°C and incubated at 60°C overnight (16 h). After addition of ligase, the mixture was incubated at 54°C for 15 min. Ligase was subsequently inactivated at 98°C for 5 min. In a next step, 10 *μ*L was transferred to PCR mix containing PCR buffer, deoxynucleotide triphosphates, Taq polymerase, and one unlabeled and one carboxyfluorescein-labeled PCR primers, which are complementary to the universal primer sequence. The PCR reaction was carried out for 33 cycles (30 sec at 95°C, 30 sec at 60°C, and 60 sec at 72°C). The fragments were analyzed on an ABI prism 3130 DNA Genetic Analyzer (Applied Biosystems).

## Results

In the present report, a large study of FA Tunisian patients was conducted by genotyping followed by MLPA. Haplotype analysis showed that among the 74 unrelated families, 78 patients from 62 families were homozygous for at least three markers (D16S3026, D16S3121 and D16S3407), so likely mutated in *FANCA* gene, eight patients from eight families were heterozygous with haplotypes disfavouring link to *FANCA* gene while the remaining four were noninformative.

**Figure 1:**
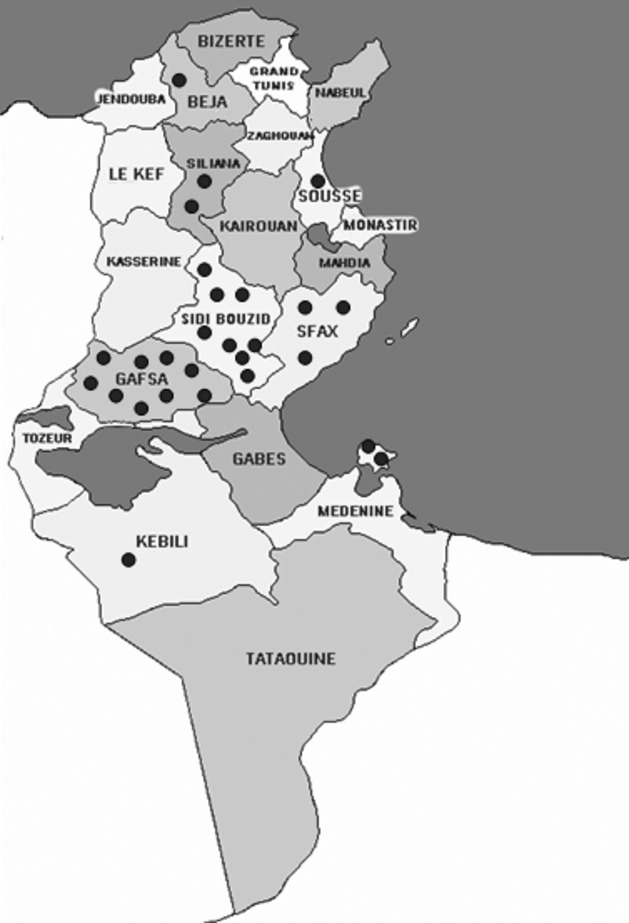
Geographic distribution of the founder mutation in *FANCA* gene in Tunisia. Eighty-five percent of the families with exon 15 founder deletion in *FANCA* gene are from southern Tunisia especially from Sidi Bouzid and Gafsa regions. Families are represented by points.

The same genetic haplotype (226-95-218-132) was shared by 42 patients (54%) belonging to 26 unrelated families. Geographic distribution of these families showed that 85% are from southern Tunisia especially from Sidi Bouzid and Gafsa regions, thus suggesting a founder effect (Fig. 1).

This founder haplotype was identified for five additional patients (GF68/09, GF53/09, GF77/09, GF07/11, and SFA6) at a heterozygous state. Cytogenetic analysis using MMC was performed for four of them. Three patients (GF53/09, GF77/09, and GF07/11) exhibited chromosomal breaks and exchanges typically found in FA cells while for the last patient (GF68/09), MMC-sensitivity tests showed inconclusive results with 52% of aberrant cells. These findings suggest a somatic mosaicism state.

**Figure 2:**
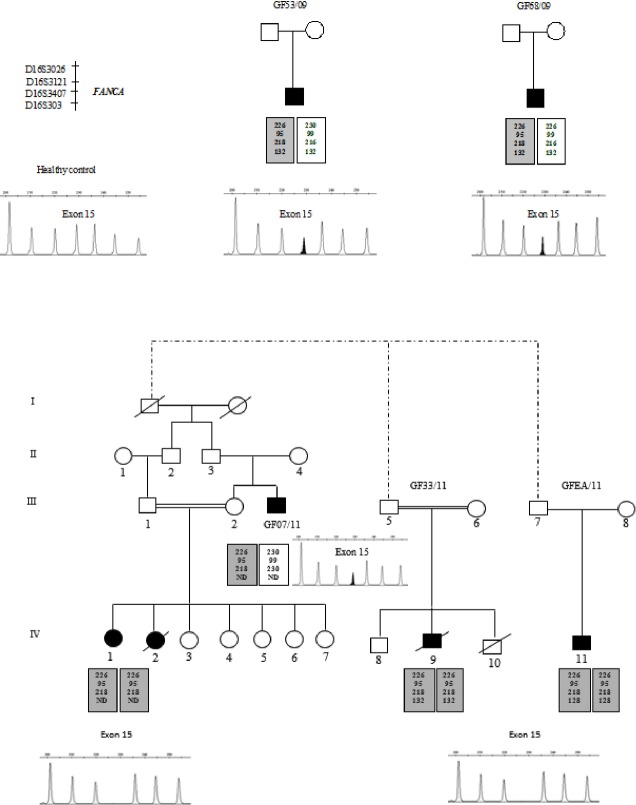
Pedigrees, haplotype analysis and results of multiplex ligation-dependent probe amplification (MLPA) assay for *FANCA* gene. GF53/09, GF68/09 and GF07/11 patients are compound heterozygous as they bear the founder haplotype at a heterozygous state and MLPA analysis show exon 15 deletion in one allele. GF33/11 and GFEA/11 patients present the founder haplotype at a homozygous state and total absence of the peak corresponding to exon 15 was noted in MLPA profile. MLPA profile for the control individual shows absence of any copy number changes.

For patients having the founder haplotype at a homozygous state, molecular analysis by MLPA revealed the presence of a total deletion of exon 15. In addition, for patients having founder haplotype at a heterozygous state, the MLPA analysis showed the deletion of exon 15 in one allele (Fig. 2).

This mutation was not found in the patients having other private haplotypes confirming that exon 15 deletion is associated only to the founder haplotype.

These results allowed us to perform a prenatal diagnosis for a consanguineous FA Tunisian family having the founder haplotype (unpubl. data).

## Discussion

Although FA is classified among rare disorders worldwide, it occurs with a relatively high frequency in Tunisia. More than 118 families with at least one affected sibling have been recorded in the TFAR (Hadiji Mseddi et al. 2012). A similar situation has been observed in some ethnic groups, such as Spanish Gypsies (Callén et al. 2005), Ashkenazi Jews (Whitney et al. 1993), and Afrikaners from South Africa (Tipping et al. 2001). All are resulting from a founder mutation. For example, founder mutations in the *FANCA* gene have been found in several populations including the South African Afrikaners, Spanish Gypsies (*FANCA* c.295C>T) (Callén et al. 2005), Moroccan Israeli Jews and in Brazilian FA patients (*FANCA* 3788-3790del mutation) (Magdalena et al. 2005). Furthermore, sub-Saharan blacks and Japanese carry founder mutations in the *FANCG* gene. In addition, *FANCC* c.456 + 4A *> *T (also known as IVS4 + 4A *> *T) is a previously identified founder mutation in the *FANCC* gene in the Ashkenazi Jewish population (Whitney et al. 1993). while *FANCC c.67delG* was identified in the Dutch and Canadian Manitoba Mennonite (de Vries et al. 2012).

A preliminary study on FA Tunisian patients has shown the presence of a founder haplotype in 12 families originating from southern Tunisia (Bouchlaka et al. 2003). This work has extended the study to 40 supplementary patients reaching a total number of 95 patients. Haplotype analysis showed the presence of a common haplotype (226-95-218-132) shared by 42 patients, mostly originating from the South of Tunisia. The high incidence of FA in southern Tunisia is likely due to an ancestral founder mutation. Indeed, we have demonstrated that this haplotype is constantly associated to exon 15 deletion in *FANCA* gene. This mutation was present at a frequency of 54% among FA-A patients (42 patients were homozygous and four were compound heterozygous).

A recent study on founder mutation in Tunisia (Romdhane et al. 2012) has shown that some of them had a specific geographic distribution. This was the case for example for the p.G1483D and p.R1763X mutations in the *COL7A1* gene in patients with Dystrophic Epidermolysa Bullosa originating from Sidi Bouzid and Kasserine, respectively. These mutations were also reported among Kuwaiti patients (Almaani et al. 2011). They likely arose in the Middle East and were introduced in Tunisia following Banu-Hilal invasions that settled in the Maghreb in the 11th century.

Based on these data, FA patients with exon 15 deletion originated from the city of Sidi Bouzid, that share a common genetic background with Middle Eastern populations, we propose to screen for this mutation patients from Arab peninsula. This mutation should be also screened for in North African patients. Indeed, FA cases of Maghrebian origin living in France share the exon 15 deletion (J. Soulier and D. S. Lyonnet, unpubl. data).

Having a founder mutation specific to the Tunisian population allowed to propose genetic counseling for the investigated families and prenatal screening of FA in Tunisia.

For the remaining 32 families likely related to *FANCA* gene, homozygosity mapping revealed several different haplotypes suggesting genetic heterogeneity in the mutation spectrum of FA in Tunisia.

In conclusion, the data presented here strongly suggest that the high incidence of FA in Tunisia is due to a founder ancestral mutation in *FANCA* gene. This mutation is probably common in the North African and Middle Eastern (MENA) region. Taking into account geographic, historical and migratory events, we hypothesize that this mutation could be shared among other patients from MENA region.

Due to limited resources in some countries and poor access to cell culture facilities, prioritization of mutation screening by direct detection of exon 15 deletion could provide a rapid and simple tool for molecular diagnosis of FA in these regions.

## Appendix

F. Talmoudi, O. Kilani, W. Ayed, A. Amouri, Department of Histology and Cytogenetics, Institut Pasteur de Tunis, Tunis, Tunisia; O. Messaoud, M. Ben Rekaya, H. Yacoub, S. Abdelhak, Laboratory of Biomedical Genomics and Oncogenetics, Institut Pasteur de Tunis, Tunis, Tunisia; L. Kammoun, S. Hdiji, M. Elloumi, Haematology Department, Hedi Chaker University Hospital, Sfax, Tunisia; L. Torjemane, A. Lakhal, N. Ben Abdeljelil, S. Ladeb, T. Ben Othman, Department of Haematology and Transplantation, National Bone Marrow Transplantation Centre, Tunis, Tunisia; M. Ouederni, F. Mellouli, M. Bejaoui, Department of Pediatric Immuno-Haematology, National Bone Marrow Transplantation, Tunis, Tunisia; L. Aissaoui, Y. Ben Abdennebi, H. Ben Abid, Hematology Department, Aziza Othmana Hospital, Tunis, Tunisia; S. Mougou, H. El Ghezal, A. Saad, Cytogenetic Department, Farhat Hached Hospital, Sousse, Tunisia; Y. Ben Youssef, A. Khelif, Hematology Department, Farhat Hached Hospital, Sousse, Tunisia; F. Amri, Paediatric department, Ibn El Jazzar Hospital Kairouan, Tunisia; M. Hachicha, Paediatric Department, Hedi Chaker Hospital, Sfax Tunisia; M. Frikha, J. Feki, Medical Carcinology Department, Habib Bourguiba Hospital, Sfax, Tunisia; F. Fakhfakh, Laboratory of Human Molecular Genetics, Faculty of Medicine, Sfax Tunisia; H. Bellaj, Intelligent Control, design and Optimization of complex Systems, National Engineering School of Sfax, Tunisia; M. Meddeb, Laboratory of Medical Genetics, Tunis, Tunisia; I. Safra, Department of Hematology, Institut Pasteur de Tunis, Tunisia; S. Hmida, National Blood Transfusion Center, Tunis, Tunisia.
